# Glycine restores the sensitivity to antibiotics in multidrug-resistant bacteria

**DOI:** 10.1128/spectrum.00164-24

**Published:** 2024-06-18

**Authors:** Cesira Giordano, Simona Barnini

**Affiliations:** 1 Microbiology Unit, Azienda Ospedaliero-Universitaria Pisana, Pisa, Italy; London Health Sciences Centre, London, Ontario, Canada

**Keywords:** glycine, checkerboard, pathogens, colistin, cefiderocol, meropenem

## Abstract

The management of infections caused by multiresistant bacteria has become of fundamental importance for any medical practice. Glycine is the most common and the simplest non-essential amino acid in humans. Glycine is very effective in improving health and supporting growth and wellbeing of humans and animals. Instead, for many bacteria, high concentrations of glycine induce lysis or deep morphological alterations. The effect of glycine on multidrug resistant (MDR) microorganisms has not yet been extensively researched. The present study was conducted 1) to establish the effect of glycine on different nosocomial pathogens isolated during routine diagnostic investigations; 2) to determine the minimum inhibitory concentration of glycine and the type of activity performed (bacteriostatic or bactericidal) on representative isolates; 3) to test the interaction between glycine and meropenem, cefiderocol, or colistin. The data reported here show a dose-dependent activity of glycine on bacteria and its bactericidal activity on MDR bacteria. Furthermore, we found that the action of glycine restores *in vitro* the susceptibility of multiresistant nosocomial pathogens to the tested antibiotics.

**IMPORTANCE:**

Antimicrobial resistance is a constantly growing concern throughout the world, and Italy is among the Western countries where antimicrobial resistance is most widespread. In Tuscany, carbapenemase-producing *Enterobacterales* are now even endemic. In this study, we challenged some resistant bacteria with a well-known molecule, glycine, the antibacterial properties of which have been known since the past century. This study could bring new insights into combining antibiotics with the simplest of all amino acids. The restoration of sensitivity to the aforementioned antibiotics by a natural compound, already used for clinical purposes, is of extreme importance in an era of proliferation of multiresistant bacteria. The *in vivo* use of this amino acid in evaluating its effectiveness against infections should be investigated. The low cost of this molecule can also make it easy to use even in low-income countries.

## INTRODUCTION

Antimicrobial resistance is a serious problem today, for all branches of medicine and hospital activity, with the well-founded prospect of further worsening in the near future (1), being constantly growing in Europe and in general throughout the world (2, 3). Italy is among the countries where antimicrobial resistance is widespread, and in Tuscany, carbapenemase-producing *Enterobacterales* are now endemic: in our hospital, a tertiary care university center, we have found extremely resistant strains, such as metallo-β-lactamases (MBL)-producing *Klebsiella pneumoniae* (4). Any molecule that can contribute to the pharmacological treatment of patients with infections caused by resistant microorganisms is welcome in this gloomy scenario. In this study, we tested some bacterial species with various types of resistance with a well-known molecule, glycine, the study of which concerning its antibacterial properties was abandoned before the worldwide diffusion of antimicrobial resistance. Just a century ago, Wyon and McLeod (5), while studying bacterial nutrition, noted that although many reports emphasized the importance of amino acids in promoting bacterial growth, nobody had noted an apparent paradox: an excess of some amino acids had an inhibitory effect on the growth of different bacterial species. In 1948, Maculla and Cowles (6) reported the lysis of bacterial cells after the addition of glycine to broth cultures and suggested the interference of glycine on unknown enzymatic processes. In 1943, Snell and Guirad (7) reported the inhibition of *Streptococcus lactis* growth by glycine and its counteraction by alanine, but not by other amino acids, and in 1951, Gordon and colleagues (8) described the kinetics of the lysis of *Bacterium coli* by glycine, suggesting that the process of lysis could be a chemical reaction and not a physical process. Fung and Winter (9) referred to different effects of both penicillin and glycine on cell wall glycopeptides of *Vibrio fetus*, supporting the contention that glycine in excessive concentrations inhibits the addition of the initial alanine to uridine diphosphate-acetylmuramic acid (UDPMurNAc), as already reported by Strominger and Birge in 1965 (10). Afterward, there were extensive studies carried out by Hishinuma and collegues (11) (1971): they proved the effects of glycine on different bacterial species, both Gram-positive and Gram-negative, aerobic and anerobic bacteria, and reported inhibition of bacterial growth by glycine as due to inhibition of UDPMurNAc-L-alanine synthetase (L-alanine adding enzyme), which incorporated glycine into UDPMurNAc, instead of L-alanine, thus causing a blockage in the construction of the bacterial wall. The effects of glycine on the bacterial wall is similar to those of penicillins and added to these, but the site of action is different since glycine and penicillin give rise to different products in the treated cells (9). Minami *et al.* (12) (2004) conducted a study to determine the effect of glycine on *Helicobacter pylori* and to propose a new therapy for *H. pylori* eradication. They showed that the inhibition of proliferation of *H. pylori* was dependent on glycine concentration; in addition, they suggested a synergistic effect between glycine and amoxicillin, acting on peptidoglycans. Cell wall synthesis involves a number of available active enzymes, such as DD- and DL-carboxypeptidases, which are essential for the formation of cell wall-bound peptidoglycans. It was suggested (12) that the modifying effect of glycine on cell wall synthesis is not only due to the inhibition of DL-carboxypeptidases but also of DD-carboxypeptidases, which are required for the synthesis of cross-linked peptidoglycan, increasing the antimicrobial efficacies of β-lactam antibiotics (13). Since 2010, more and more MDR bacterial species have spread all over the world, and also in our region, Tuscany, Italy (14). The infections they cause are often difficult to treat, and the difficulties are even greater when it comes to sepsis or septic shock, given the need to establish adequate antibiotic therapy as soon as possible. Triggered by the increasing prevalence of MDR isolates, the past decade has seen a great expansion not only in the development of MDR bacterial detection techniques but also in antimicrobial compound research (15, 16). Although new antimicrobials have been recently approved or are in the pipeline showing promising results, the appropriate use of these agents and the need for new compounds is still required (17, 18). In the present study, we focused on glycine, the simplest of amino acids. The antibacterial activity of glycine and the interaction of glycine plus clinically relevant antimicrobial agents (meropenem, cefiderocol, and colistin) against MDR nosocomial pathogens were evaluated.

## MATERIALS AND METHODS

### Bacterial strain selection

Bacterial strains were isolated during the laboratory routine practice from patients admitted to the Pisa University Hospital (Azienda Ospedaliero-Universitaria Pisana - AOUP) and randomly selected for further investigation. Strains were taken from remnants of patients' standard samples and used anonymously. For this type of study, no written informed consent was required. Various biological samples were cultured on common isolation media in routine diagnostics and incubated at 37°C. Colonies were identified using MALDI-TOF MS (Bruker Daltonik GmbH, Bremen, Germany) according to the manufacturer’s instructions. Antimicrobial susceptibility tests were performed using broth microdilution methods (SensiTitre-Thermo Fisher Scientific, MA, USA, or MICRONAUT AST-MERLIN Diagnostika GmbH) according to the manufacturer’s instructions. The minimal inhibitory concentration (MIC) results were interpreted according to European Committee on Antimicrobial Susceptibility Testing (EUCAST) guidelines. An initial molecular screening was performed using the GeneXpert System (Cepheid, Sunnyvale, CA, USA), with the Xpert-CARBA and the Xpert*vanA/vanB* tests. These tests are based on real-time PCR, which identifies, through the use of specific primers, specific resistance genes. It is mandatory in AOUP to store multidrug-resistant isolates after routine clinical microbiological testing (identification and antimicrobial susceptibility testing) has been completed. Biological samples were cultured on common isolation media, and colonies of interest were frozen in brain heart infusion broth supplemented with 10% glycerol for any future investigations (19). Selected bacterial strains were whole-genome-sequenced. Total genomic DNA was extracted from fresh cultures using an UltraClean Microbial DNA Isolation Kit (MoBio Laboratories, Carlsbad, California, United States) according to the manufacturer’s instructions. The concentration and purity of the extracted DNA were determined with a Qubit 2.0 fluorometer using the dsDNA BR Assay Kit (Life Technologies, Carlsbad, California, United States). DNA libraries were prepared using the Nextera kit (Illumina Inc., San Diego, California, United States), according to the manufacturer’s instructions and were then run on a MiSeq system (Illumina Inc.) to generate 250-bp paired-end reads.

### Reference strains and accession numbers

*Klebsiella pneumoniae* ATCC 1705, *K. pneumoniae* ATCC 1706, and *E. faecalis* ATCC 51922 were used as reference strains. Bacterial sequences were submitted in the study PRJNA1090287.

### Glycine MIC

MIC values of glycine (A.C.E.F. s.p.a) were determined in duplicate using broth microdilution susceptibility tests against 154 nosocomial pathogens isolated in clinical routine practice. In detail, 10 isolates of *Escherichia coli*, 10 of *Proteus mirabilis*, four of *Proteus vulgaris*, six of *Morganella morganii*, one of *Raoultella ornithinolytica*, 60 of *Klebsiella pneumoniae*, seven of *Acinetobacter baumannii*, four of *Klebsiella oxytoca*, 10 of *Pseudomonas aeruginosa*, 10 of *Stenotrophomonas maltophilia*, 10 of *Enterobacter cloacae*, five of *Enterobacter aerogenes*, six of *Citrobacter freundii*, 10 of *Serratia marcescens,* one *Yersinia enterocolitica*, and one *Enterococcus faecalis* VanA producer were screened. Brieﬂy, serial dilutions of glycine were prepared in 96-well microtiter plates (Sarstedt, Nümbrecht, Germany) with cation-adjusted Mueller–Hinton broth (MERLIN Diagnostika GmbH). For each strain, seven different concentrations of glycine were assessed: 0.28 M, 0.32 M, 0.36 M, 0.40 M, 0.44 M, 0.48 M, and 0.52 M. A well without glycine was used as negative control (final volume of each well was 100 µL). For each strain, a 0.5 McFarland suspension was prepared, and then serial dilutions were performed to obtain an initial inoculum of 5*10^5^ CFU/mL. The MIC was taken as the lowest concentration of glycine, resulting in the complete inhibition of visible growth after 18 hours of incubation at 37°C. The bacteriostatic and bactericidal activity was determined using a modiﬁed assay by Aumeeruddy–Elalﬁ and colleagues (20). In detail, 10 µL of broth from the well where no visible bacterial growth was observed in the previously cited MIC assay, corresponding to the MIC value, was inoculated onto sterile blood agar plates and incubated for 18 hours. Growth of bacteria indicates bacteriostasis, while no growth indicates bactericidal effects.

### Combination of glycine with selected antibiotics

The strains *K. pneumoniae* ATCC 1705, *K. pneumoniae* ATCC 1706, and *E. faecalis* ATCC 51922 were used as references to measure the interaction of glycine with meropenem, cefiderocol, and colistin by using the microbroth checkerboard assay. Synergistic activity between glycine and meropenem (Glentham, Life Science) was assessed for 10 clinical isolates of *K. pneumoniae* KPC producers (KPC) and 30 *K*. *pneumoniae* New Delhi MBL producers, previously whole-genome-sequenced (19, 21–23). The combination of glycine and cefiderocol (Shionogi & Company) was evaluated in five cefiderocol-resistant-NDM carbapenemase-producing *Klebsiella pneumonia* (accession numbers SAMN40561758–SAMN40561762, study PRJNA1090287) and five cefiderocol-resistant *Acinetobacter baumannii* (accession numbers SAMN40561763–SAMN40561767, study PRJNA1090287). The interaction between glycine and colistin sulfate (Discovery Fine Chemicals) was assessed for two colistin-resistant *Acinetobacter baumannii* (accession numbers SAMN40561772–SAMN40561773, study PRJNA1090287) and four colistin-resistant *Klebsiella pneumoniae* (two plasmid-mediated and two chromosome-mediated colistin resistance) (4, 24).

Each well was filled with Muller–Hinton broth (MERLIN Diagnostika GmbH) containing the specific antibiotic at 10 doubling dilutions and glycine (A.C.E.F. s.p.a) dispensed in a checkerboard manner. In detail, serial dilutions of both antibiotic and glycine were prepared in 96-well microtiter plates (Sarstedt, Nümbrecht, Germany) with cation-adjusted Mueller–Hinton broth (MERLIN Diagnostika GmbH). For each strain, a 0.5 McFarland suspension was prepared, and then serial dilutions were performed to obtain an initial inoculum of 5*10^5^ CFU/mL. Different concentrations of glycine (0.28 M, 0.32 M, 0.36 M, 0.40 M, 0.44 M, 0.48 M, and 0.52 M) were evaluated in combination with meropenem ranging from 64 mg/L to 0.06 mg/L, cefiderocol ranging from 128 mg/L to 0.12 mg/L, and colistin ranging from 64 mg/L to 0.06 mg/L. In each plate, one well was used as the growth control, without the antibiotic or glycine. The MIC was taken as the lowest concentration of the antibiotic and glycine in combination, resulting in the complete inhibition of visible growth after 18 hours of incubation at 37°C. The fractional inhibitory concentration index (FiCi) was used to interpret the checkerboard assay and was calculated using the Loewe Additivity method (25): FiCi = FiC of drug A + FiC of drug B. The FiCs of drugs A and B were calculated using the following formulas: FiC of drug A = MIC of drug A in combination/MIC of drug A alone; FiC of drug B = MIC of drug B in combination/MIC of drug B alone. Therefore, an FiCi <0.5 indicates that doses A and B producing a given effect in combination are lower than the expected doses from additivity and can hence be directly interpreted as synergy. FiCi in the 0.5 to 1 range are considered to be additive. FiCi from 1 to 4 are defined as indifferent, while those over 4 are antagonistic (26, 27).

### Time kill curves

For representative isolates, growth curves were analyzed in real-time in triplicate using the HB&L instrument (Alifax S.r.l, Italy). The instrument is certified to assess growth curves based on a light-scattering technique that reliably detects microbial growth in fluid samples. It calculates real-time growth curves and bacterial counts (CFU/mL) following a patented algorithm (24, 28). Growth curves were performed at different concentrations of glycine (0.28 M, 0.32 M, 0.36 M, 0.40 M, 0.44 M, 0.48 M, and 0.52 M) in cation-adjusted Mueller–Hinton broth (MERLIN Diagnostika GmbH) in a final volume of 2 mL. For each strain, a 0.5 McFarland suspension was prepared, and then serial dilutions were performed to obtain an initial inoculum of 5*10^5^ CFU/mL. The exact inocula were confirmed by plating the serial dilutions of cultures. After a blank reading to set the analytical zero, the scattering units were measured every 5 minutes for 24 hours at 37°C, detecting only viable, replicating bacteria. Control curves, without glycine, were performed and used as comparators.

## RESULTS

### Characteristics of isolates

The 154 clinical isolates used to assess the range of glycine activity had various antibiotic susceptibility profiles. *K. pneumoniae* clinical isolates, selected by the checkerboard method, were MDR strains. Particularly, the MIC of meropenem ranged from 16 to above 64 mg/L. They belonged to different sequence types: one of the KPC-Kp isolates belonged to ST307 and nine strains belonged to ST512; one of the NDM-Kp isolates belonged to ST307, and 29 NDM-Kp belonged to ST147. In all KPC-Kp isolates, the transposon *Tn4401a* harboring the carbapenemase gene *bla*_KPC-3_ carried by the *pKpQIL* plasmid was detected. In the isolates belonging to ST307, the CTX-M-15 extended-spectrum β-lactamase was also identified. In addition, we detected different combinations of β-lactamase genes encoding for OXA-1, OXA-9, SHV-11, SHV-28, TEM-1A, and TEM-1B. The NDM-Kp showed uniform resistance to extended-spectrum cephalosporins, carbapenems, aztreonam, fluoroquinolones, and the novel β-lactamase inhibitor combinations ceftazidime–avibactam and meropenem–vaborbactam and frequent resistance to aminoglycosides. They were mostly susceptible to fosfomycin and colistin and uniformly susceptible to cefiderocol and to the aztreonam–avibactam combination. Resistome profiling revealed the constant presence of the chromosomal blaSHV-11 and of a gene encoding a truncated OmpK35. Concerning the acquired resistome, along with the *blaNDM-1* and *blaCTX-M-15* β-lactamase genes, detected for all isolates, a plethora of other acquired determinants associated with resistance to β-lactam antibiotics (*blaTEM-1, blaTEM-32, blaOXA-1,* and *blaOXA-9*) were also variably present (14, 18, 19, 22–24). *Enterococcus faecalis* was resistant to glycopeptides and harbored the *vanA* gene. *Acinetobacter baumannii* strains were MDR (29). Whole-genome sequencing analysis revealed that the *A. baumannii* isolates belong to sequence type ST2, according to the MLST Pasteur database. The isolates harbor class D and C β-lactamase genes, including *bla*_OXA23_, *bla*_OXA66_, and *bla*_ADC25_. Several genes also involved in cefiderocol resistance were investigated. A missense mutation in penicillin-binding protein-3 (PBP-3) was detected, resulting in amino acid change N235K; the *piuA* showed a frameshift that determines a premature stop codon (K384fs); the *pirA* showed a frameshift at position I522fs; the *fepA* gene was interrupted by a transposon insertion P635-ISAba125 (IS30 family) (study PRJNA926509 (18) and study PRJNA1090287).

### Phenotypic analysis of reference strains

Reference strains were tested in microdilution to check for antibiotic susceptibility. In particular, the susceptibility of KPC-Kp ATCC 1705 to antibiotics was also tested by adding glycine at concentrations of 0.125, 0.25, and 0.3M to the microdilution susceptibility panel (SensiTitre, Thermo Fisher Scientific): the results showed a dose-dependent decrease in MIC values for doripenem, imipenem, meropenem, and ceftazidime (from >8 to 2, from 8 to <1, from 16 to 2, and from 64 to 8 mg/L, respectively).

### Glycine MIC

Regarding reference strains, the MIC of glycine was 0.40 M for *K. pneumoniae* ATCC 1705, 0.44 M for *K. pneumoniae* ATCC 1706, and 1 M for *E. faecalis* ATCC 51922; MICs ranged from 0.40 m to 0.52 M for *C. freundii*; from 0.32 M to >0.52 M for *E. aerogenes*; from 0.32 M to >0.52 M for *E. cloacae*; from 0.28 M to 0.36 M for *E.coli*; from 0.40 M to 0.52 M for *K. oxytoca*; from 0.28 M to 0.52 M for *K. pneumoniae*; from 0.40 M to >0.52 M for *P. aeruginosa*; from 0.28 M to 0.48 M for *S. maltophilia*; higher or equal to 0.52 M for *P. mirabilis*; higher than 0.52 M for *M. morganii*, for *P. vulgaris*, for *R. ornithinolytica*, *Y. enterocolitica,* and for *E. faecalis* (Table 1). For KPC-Kp and NDM-Kp, MIC values ranged from 0.36 M to 0.44 M. For *A. baumannii,* MIC values ranged from 0.40 M to 0.44 M. To determine the bacteriostatic or bactericidal activity, 10 µL of Mueller–Hinton broth, corresponding to the MIC value well, was inoculated on sterile blood agar plates. For *K. pneumoniae*, after an overnight incubation at 37°C, no bacterial growth was observed for 32 clinical isolates, while, compared with the initial inoculum, a dramatic decrease in the number of viable cells was observed for the other eight clinical isolates and the two reference strains (>3 log_10_ CFU/mL), confirming the bactericidal activity of glycine. Bactericidal activity was also observed for *A. baumannii*.

**TABLE 1:** Number of the strains with different glycine MICs. M, molar

Glycin concentration	0.28 M	0.32 M	0.36 M	0.4 M	0.44 M	0.48 M	0.52 M	>0.52 M
*Citrobacter freundii*				1	1	2	2	
*Enterobacter aerogenes*		1					2	2
*Enterobacter cloacae*		1	1		1	1	5	1
*Escherichia coli*	6	1	2				1	
*Klebsiella oxytoca*				1	2		1	
*Klebsiella pneumoniae*	1	1	7	22	21	3	5	
*Acinetobacter baumannii*				4	3			
*Morganella morganii*								6
*Proteus mirabilis*							4	6
*Proteus vulgaris*								4
*Pseudomonas aeruginosa*				1			5	4
*Raoultella ornithinolytica*								1
*Serratia marcescens*								10
*Stenotrophomonas maltophilia*	2	2	1	1		4		
*Yersinia enterocolitica*								1
*Enterococcus faecalis VanA*								1

### Time kill curve analysis

**Fig 1:**
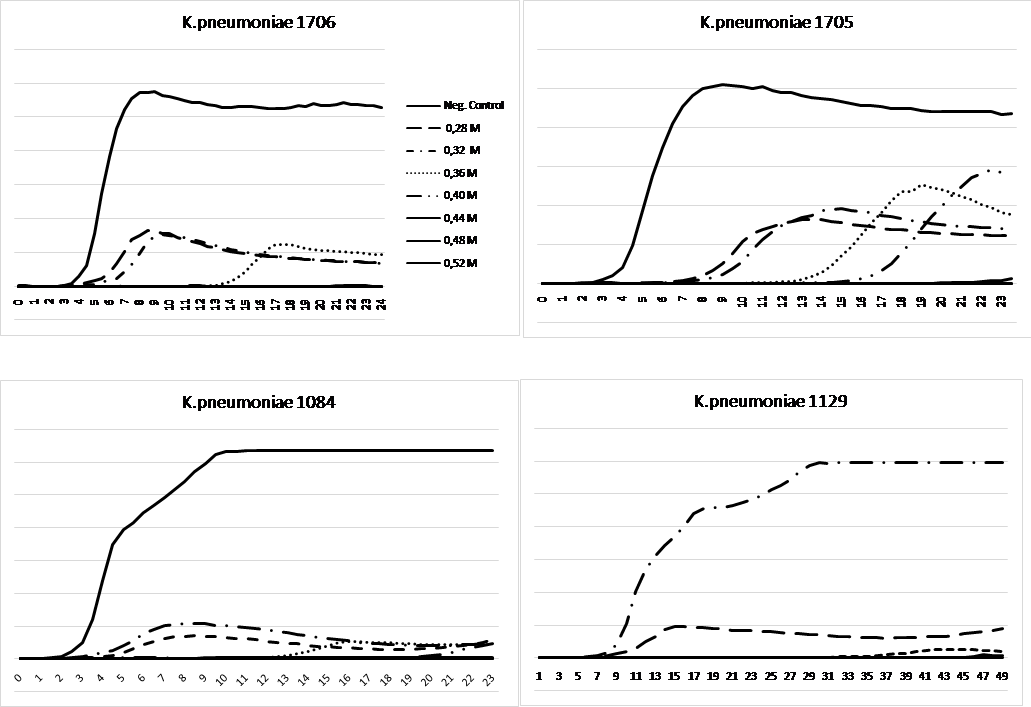
Growth curves of two *Klebsiella pneumoniae* ATCC strains, 1706 and 1705, and of two clinical isolates belonging to ST512 (1084) and to ST307 (1129) in the presence of glycine at different concentrations. In the absence of glycine, the four strains showed similar behaviors, with a lag-phase length of 3.5 hours for *K. pneumoniae* ATCC 1706, *K. pneumoniae* ATCC 1705, and *K. pneumoniae* 1129 and 3 hours for *K. pneumoniae* 1084. A complete killing was observed at a glycine concentration of 0.36 to 0.44 M.

Growth curves were analyzed using the HB&L instrument (Alifax S.r.l.) for ATCC reference strains and for two different clinical isolates of *Klebsiella pneumoniae* belonging to ST512 (*K. pneumoniae* 1084) and to ST307 (*K. pneumoniae* 1129), selected as representatives. Results confirmed broth microdilution MIC values. As shown in Fig. 1, in the absence of glycine, the four isolates showed a similar behavior, with a lag-phase length of 3.5 hours for *K. pneumoniae* ATCC 1705, *K. pneumoniae* ATCC 1706, and *K. pneumoniae* 1129 and 3 hours for *K. pneumoniae* 1084.

*K. pneumoniae* ATCC 1706 showed a complete killing at glycine MIC of 0.40 M. At glycine MIC of 0.28 M, 0.32 M, and 0.36 M, the lag phases of the isolate increased exponentially, passing from 3.5 hours in the negative control to 4 hours, 5 hours, and 13.5 hours, respectively.

*K. pneumoniae* ATCC 1705 showed a complete killing at glycine MIC of 0.44 M. At glycine MIC of 0.28 M, 0.32 M, 0.36 M, and 0.40 M, the lag phases of the isolate increased exponentially, passing from 3.5 hours in the negative control to 7.5 hours, 8 hours, 13 hours, and 15.5 hours, respectively.

*K. pneumoniae* 1084 showed a complete killing at glycine MIC of 0.44 M. At glycine MIC of 0.28 M, 0.32 M, 0.36 M, and 0.40 M, the lag phases of the isolate increased exponentially, passing from 3 hours in the negative control to 5 hours, 4.5 hours, 13.5 hours, and 20.5 hours, respectively.

*K. pneumoniae* 1129 showed a complete killing at glycine MIC of 0.36 M. At glycine MIC of 0.28 M and 0.32 M, the lag phase of the isolate exponentially increased, passing from 3.5 hours in the negative control to 3.5 hours, and 17.5 hours, respectively. These results show a dose-dependent activity of glycine and confirmed its bactericidal activity.

Comparisons of the lag-phase values with and without glycine were analyzed using Student’s t-test. In the absence of glycine, the four strains analyzed showed the same lag-phase, while the exposure to glycine for *K. pneumoniae* KPC-positive strains 1084 and 1129, compared to the ATCC 1705 (harboring the *bla*_KPC_ gene) determined statistically significant changes in the lag-phase timing.

### Effects of the combinations between glycine and the antibiotics

The effects of the combination of glycine with meropenem, cefiderocol, or colistin against MDR clinical isolates were determined. In the presence of glycine, for the reference strain *K. pneumoniae* ATCC 1705 meropenem, the MIC decreased from 4 mg/L to 0.5 mg/L. Eight KPC-Kp and four NDM-Kp isolates had meropenem MIC above 64 mg/L: in the presence of glycine, for one isolate, the meropenem MIC decreased to 0.25 mg/L; for two isolates, it decreased to 0.5 mg/L; for three isolates, it decreased to 2 mg/L; and for six isolates, it decreased to 4 mg/L. For all isolates, the combination of glycine and meropenem yielded signiﬁcant inhibition of growth. The combination of meropenem and glycine enhanced the killing effect against carbapenemase-producing *K. pneumoniae* and resulted in a dramatic decrease in growth, which was greater than that achieved with glycine or meropenem alone (Table 2). In the presence of glycine, cefiderocol MIC for cefiderocol-resistant *K. pneumoniae* decreased from 128 mg/L to 4 mg/L for one isolate, from 64 mg/L to 4 mg/L for three isolates, and from 8 mg/L to 2 mg/L for one isolate; cefiderocol MIC for cefiderocol-resistant *A. baumannii* decreased from 128 mg/L to 1 mg/L for two isolates and from 64 mg/L to 0.5 mg/L for three isolates (Table 3). In the presence of glycine, colistin MIC for colistin-resistant *A. baumannii* decreased from 64 mg/L to 2 mg/L and from 4 mg/L to 0.25 mg/L; colistin MIC for colistin-resistant *K. pneumoniae* decreased from 8 mg/L to 1 mg/L for the four isolates (Table 4). The combined effect between each of the three antibiotics and glycine showed a cooperative (additive) activity (FiCi <1).

**TABLE 2:** Checkerboard results showing glycine and meropenem MICs and the effect of their combination^*a*^

	MIC				FiC	FiC	FiCi
Isolates	Meropenem	Glycine	Mer + Gly	Gly + Mer	Mer	Gly	
	(mg/L)	(M)	(mg/L)	(M)			
*Klebsiella pneumoniae* ATCC 1705	4	0^.^40	0^.^5	0^.^28	0^.^13	0^.^70	0^.^83
*Klebsiella pneumoniae* ATCC 1706	0^.^5	0^.^44	0^.^12	0^.^28	0^.^24	0^.^46	0^.^88
*Klebsiella pneumoniae* KPC 1084	64	0^.^44	4	0^.^40	0^.^06	0^.^90	0^.^97
*Klebsiella pneumoniae* KPC 1091	64	0^.^44	2	0^.^40	0^.^03	0^.^90	0^.^93
*Klebsiella pneumoniae* KPC 1043	64	0^.^40	2	0^.^32	0^.^03	0^.^80	0^.^83
*Klebsiella pneumoniae* KPC1059	64	0^.^40	4	0^.^32	0^.^06	0^.^80	0^.^86
*Klebsiella pneumoniae* KPC1076	64	0^.^44	0^.^5	0^.^36	0^.^01	0^.^82	0^.^83
*Klebsiella pneumoniae* KPC1079	64	0^.^44	2	0^.^36	0^.^03	0^.^82	0^.^85
*Klebsiella pneumoniae* KPC1123	64	0^.^40	4	0^.^36	0^.^06	0^.^90	0^.^96
*Klebsiella pneumoniae* KPC1129	16	0^.^36	2	0^.^28	0^.^13	0^.^78	0^.^91
*Klebsiella pneumoniae* KPC1145	64	0^.^40	4	0^.^32	0^.^06	0^.^80	0^.^86
*Klebsiella pneumoniae* KPC1206	32	0^.^40	2	0^.^28	0^.^06	0^.^70	0^.^76
*Klebsiella pneumoniae* NDM 1	8	0^.^44	1	0^.^28	0^.^13	0^.^64	0^.^76
*Klebsiella pneumoniae* NDM 2	64	0^.^44	4	0^.^36	0^.^06	0^.^82	0^.^88
*Klebsiella pneumoniae* NDM 7	64	0^.^40	1	0^.^28	0^.^02	0^.^70	0^.^72
*Klebsiella pneumoniae* NDM 8	8	0^.^44	0^.^5	0^.^25	0^.^06	0^.^57	0^.^63
*Klebsiella pneumoniae* NDM 9	64	0^.^40	0^.^5	0^.^32	0^.^01	0^.^80	0^.^81
*Klebsiella pneumoniae* NDM 10	64	0^.^44	0^.^25	0^.^36	0^.^00	0^.^82	0^.^82
*Klebsiella pneumoniae* NDM 11	16	0^.^40	0^.^5	0^.^28	0^.^03	0^.^70	0^.^73
*Klebsiella pneumoniae* NDM 12	64	0^.^44	0^.^25	0^.^32	0^.^00	0^.^73	0^.^73
*Klebsiella pneumoniae* NDM 14	64	0^.^44	1	0^.^28	0^.^02	0^.^64	0^.^65
*Klebsiella pneumoniae* NDM 15	64	0^.^44	0^.^5	0^.^32	0^.^01	0^.^73	0^.^74
*Klebsiella pneumoniae* NDM 16	32	0^.^40	2	0^.^28	0^.^06	0^.^70	0^.^76
*Klebsiella pneumoniae* NDM 17	16	0^.^40	1	0^.^28	0^.^06	0^.^70	0^.^76
*Klebsiella pneumoniae* NDM 18	8	0^.^40	0^.^5	0^.^28	0^.^06	0^.^70	0^.^76
*Klebsiella pneumoniae* NDM 19	64	0^.^44	1	0^.^32	0^.^02	0^.^73	0^.^74
*Klebsiella pneumoniae* NDM 20	16	0^.^40	1	0^.^28	0^.^06	0^.^70	0^.^76
*Klebsiella pneumoniae* NDM 23	8	0^.^40	0^.^5	0^.^28	0^.^06	0^.^70	0^.^76
*Klebsiella pneumoniae* NDM 24	8	0^.^40	0^.^5	0^.^28	0^.^06	0^.^70	0^.^76
*Klebsiella pneumoniae* NDM 25	16	0^.^40	0^.^5	0^.^28	0^.^03	0^.^70	0^.^73
*Klebsiella pneumoniae* NDM 26	8	0^.^36	0^.^25	0^.^32	0^.^03	0^.^89	0^.^92
*Klebsiella pneumoniae* NDM 27	8	0^.^40	1	0^.^28	0^.^13	0^.^70	0^.^83
*Klebsiella pneumoniae* NDM 28	32	0^.^44	1	0^.^28	0^.^03	0^.^64	0^.^67
*Klebsiella pneumoniae* NDM 29	32	0^.^36	0^.^25	0^.^32	0^.^01	0^.^89	0^.^90
*Klebsiella pneumoniae* NDM 30	16	0^.^40	1	0^.^32	0^.^06	0^.^80	0^.^86
*Klebsiella pneumoniae* NDM 31	64	0^.^44	2	0^.^36	0^.^03	0^.^82	0^.^85
*Klebsiella pneumoniae* NDM 32	8	0^.^40	0^.^25	0^.^32	0^.^03	0^.^80	0^.^83
*Klebsiella pneumoniae* NDM 38	4	0^.^40	0^.^12	0^.^32	0^.^03	0^.^80	0^.^83
*Klebsiella pneumoniae* NDM 39	16	0^.^36	0^.^25	0^.^28	0^.^02	0^.^78	0^.^79
*Klebsiella pneumoniae* NDM 40	16	0^.^36	0^.^5	0^.^28	0^.^03	0^.^78	0^.^81
*Klebsiella pneumoniae* NDM 41	32	0^.^40	0^.^5	0^.^28	0^.^02	0^.^70	0^.^72
*Klebsiella pneumoniae* NDM 42	32	0^.^36	0^.^5	0^.^28	0^.^02	0^.^78	0^.^79

^*a*^ Mer, meropenem; Gly, glycine; FiC fractional inhibitory concentration; i, index.

**TABLE 3:** Checkerboard results showing glycine and colistin MICs and the effect of their combination^*a*^

	MIC				FiC	FiC	FiCi
Isolates	COL	Glycine	COL + Gly	Gly +COL	COL	Gly	
	(mg/L)	(M)	(mg/L)	(M)			
*Klebsiella pneumoniae* COLR 1	8	0.52	1	0.4	0.13	0.77	0.89
*Klebsiella pneumoniae* COLR 2	8	0.52	1	0.4	0.13	0.77	0.89
*Klebsiella pneumoniae* COLR 3	32	0.48	0.5	0.36	0.02	0.75	0.77
*Klebsiella pneumoniae* COLR 4	32	0.48	0.5	0.36	0.02	0.75	0.77
*Acinetobacter baumannii* COLR 1	4	0.4	0.25	0.28	0.06	0.7	0.76
*Acinetobacter baumannii* COLR 2	64	0.4	0.25	0.36	0	0.9	0.9

^*a*^ COL, colistin; R, resistant; Gly, glycine; FiC, fractional inhibitory concentration; i, index.

**TABLE 4:** Checkerboard results showing glycine and cefidrocol MICs and the effect of their combination^*a*^

	MIC				FiC	FiC	FiCi
Isolates	CFD	Glycine	CFD + Gly	Gly +CFD	CFD	Gly	
	(mg/L)	(M)	(mg/L)	(M)			
*Klebsiella pneumoniae* CFDR 1	128	0.4	1	0.28	0.01	0.7	0.71
*Klebsiella pneumoniae* CFDR 2	128	0.44	1	0.32	0.01	0.73	0.74
*Klebsiella pneumoniae* CFDR 3	64	0.4	0.5	0.28	0.01	0.7	0.71
*Klebsiella pneumoniae* CFDR 4	64	0.44	1	0.28	0.02	0.64	0.65
*Klebsiella pneumoniae* CFDR 5	64	0.44	1	0.28	0.02	0.64	0.65
*Acinetobacter baumannii* CFDR 6	128	0.44	4	0.32	0.03	0.73	0.76
*Acinetobacter baumannii* CFDR 7	64	0.44	8	0.32	0.13	0.73	0.85
*Acinetobacter baumannii* CFDR 8	64	0.4	4	0.32	0.06	0.8	0.86
*Acinetobacter baumannii* CFDR 9	8	0.44	2	0.32	0.25	0.73	0.98
*Acinetobacter baumannii* CFDR 10	64	0.4	8	0.28	0.13	0.7	0.83

^*a*^ CFD, cefiderocol; R, resistant; Gly, glycine; FiC, fractional inhibitory concentration; i, index.

## DISCUSSION

The peculiar effects of glycine on bacteria have been known since 1943 (6, 8, 13, 30, 31). Glycine has a crucial function in cytoprotection, growth, development, metabolism, immune response, and survival of humans and other animals. Biochemical studies on animal models proved that glycine is synthesized from threonine, choline, and serine. Glycine acts as a precursor for several key metabolites of low molecular weight such as creatine, glutathione, heme, purines, and porphyrins. Degradation of glycine in humans is done via three pathways: (1) D-aminoacid oxidase converting glycine into glyoxylate, (2) serine hydroxymethyltransferase converting glycine into serine, and (3) deamination and decarboxylation by the glycine cleavage enzyme system. Focusing on clinical aspects, it was reported that oral supplementation of glycine in animal models can be very effective in protecting the alcohol-induced hepatotoxicity since glycine is able to optimize the activity of a variety of enzymes (32). From *in vivo* studies, it was demonstrated that certain cancers, schizophrenia, stroke, wounds, ulcers, organ transplantation failures, several intestinal and stomach disorders, and some of the rare inherited metabolic disorders can be prevented or cured by oral or intravenous glycine supplementation in a dose-dependent manner or by direct application to the skin. In addition, experimental studies supported the hypothesis that glycine also has anti-inflammatory and organ-protective effects in an animal model of trauma, shock, and sepsis (33, 34). More recently, a new therapeutic approach was suggested for *H. pylori* eradication since it was demonstrated that the inhibition of proliferation of *H. pylori* was dependent on the glycine concentration and that there was a synergistic effect with amoxicillin/clavulanate (12). The mechanism of action of glycine has already been investigated and involves the synthesis of peptidoglycan components (5–10). The morphological effects of glycine on different bacterial species are similar: there is an elongation of the bacterial body accompanied by a swelling of the cell. Cell wall synthesis includes a number of available active enzymes such as DD- or DL-carboxypeptidases, which are important for the formation of cell wall-bound peptidoglycans from UDP-MUrNAc-tetra-peptides and tri-peptides, respectively. Because glycine and D-aminoacids inhibit LD-carboxypeptidase, their modifying effect on cell wall synthesis is explained mainly in this way. Other enzymes also involved in cell wall biosynthesis may also be affected by aminoacids (35). One example is the L-ala-adding enzyme (UDP-MurNac-L-ala-sythetase), which is strongly inhibited by glycine (11). Presumably, this enzymatic system is the main target of the combination of glycine and β-lactams, thus explaining the influence of glycine on bacterial reactivity to β-lactams. Beta-lactam antibiotics are capable of inhibiting not only DL-carboxypeptidases but also DD-carboxypeptidases, which are required for the synthesis of cross-linked peptidoglycans. The FiCis here reported support the additivity role between glycine and beta-lactams on multidrug-resistant bacteria. The bactericidal activity of glycine was established on multidrug-resistant bacteria. As shown in the time-kill curves assay, without glycine, all the strains behave in the same manner, while, by adding glycine, strain growth was significantly different (*P* < 0.05). This shows that the action mode of glycine is specific for each strain and affects the replication process. The cooperative effect of glycine, understood as the cause of the antimicrobial efficacy of β-lactam antibiotics, is mainly due to its inhibition of this enzymatic system (12) and is so effective as to cause the phenotypic restoration of susceptibility to the antibiotics tested in all the MDR bacterial strains examined.

In conclusion, the data reported here show a dose-dependent activity of glycine on bacteria and confirmed the bactericidal activity of this amino acid also on MDR bacteria. In addition, we demonstrated a positive interaction between meropenem, cefiderocol, or colistin in combination with glycine against MDR nosocomial pathogens. The restoration of susceptibility to well-known antibiotics by a natural compound, already used for clinical purposes, is certainly of utmost importance in an era of proliferation of MDR microorganisms, and the *in vivo* employment of this amino acid in humans should be further investigated to evaluate its efficacy against infection and/or colonization. Low-cost therapies are needed above all for low-income countries to fight infections and antibiotic resistance, given the worrying predictions for the near future (36–39).
